# Design and Evaluation of Anti-SARS-Coronavirus Agents Based on Molecular Interactions with the Viral Protease

**DOI:** 10.3390/molecules25173920

**Published:** 2020-08-27

**Authors:** Kenichi Akaji, Hiroyuki Konno

**Affiliations:** 1 Department of Medicinal Chemistry, Kyoto Pharmaceutical University, Yamashina, Kyoto 607-8414, Japan; 2 Hamari Chemicals, Ltd., Suminoe-ku, Osaka 559-0034, Japan; 3 Chemical Engineering and Biochemical Engineering, Yamagata University, Yonezawa, Yamagata 992-8510, Japan; konno@yz.yamagata-u.ac.jp

**Keywords:** corona-virus, SARS, protease, inhibitor

## Abstract

Three types of new coronaviruses (CoVs) have been identified recently as the causative viruses for the severe pneumonia-like respiratory illnesses, severe acute respiratory syndrome (SARS), Middle East respiratory syndrome (MERS), and corona-virus disease 2019 (COVID-19). Neither therapeutic agents nor vaccines have been developed to date, which is a major drawback in controlling the present global pandemic of COVID-19 caused by SARS coronavirus 2 (SARS-CoV-2) and has resulted in more than 20,439,814 cases and 744,385 deaths. Each of the 3C-like (3CL) proteases of the three CoVs is essential for the proliferation of the CoVs, and an inhibitor of the 3CL protease (3CL^pro^) is thought to be an ideal therapeutic agent against SARS, MERS, or COVID-19. Among these, SARS-CoV is the first corona-virus isolated and has been studied in detail since the first pandemic in 2003. This article briefly reviews a series of studies on SARS-CoV, focusing on the development of inhibitors for the SARS-CoV 3CL^pro^ based on molecular interactions with the 3CL protease. Our recent approach, based on the structure-based rational design of a novel scaffold for SARS-CoV 3CL^pro^ inhibitor, is also included. The achievements summarized in this short review would be useful for the design of a variety of novel inhibitors for corona-viruses, including SARS-CoV-2.

## 1. Introduction

The term coronavirus (CoV) is derived from the crown-like spikes on the surface of the virus. CoVs are enveloped positive-strand RNA viruses that infect various vertebrates, including humans. In the 1960s, two human coronaviruses, the human alpha coronavirus 229E (HCoV-229E) and the human beta coronavirus OC43 (HCoV-OC43) were discovered as causative agents of disorders such as the common cold or respiratory illnesses of mild to moderate severity [1,2]. Additionally, two human coronaviruses, the alpha coronavirus NL63 [3,4,5] and the beta coronavirus HKU1 [6,7], were then identified in 2004 and 2005. At the same time, a contrasting new human beta coronavirus causing life-threatening illnesses (severe acute respiratory syndrome (SARS)-CoV) was identified in 2003 [8,9,10]. SARS spread rapidly worldwide from its likely origin in southern China; the subsequent SARS epidemic involved approximately 8500 patients, with more than 800 fatalities. After nearly a decade, in 2012, a new respiratory illness similar to SARS, named Middle East respiratory syndrome (MERS), affected more than 1800 patients with a fatality rate of 36% [11,12]. Even after these pandemics, no effective therapy has been developed for CoVs infections, and the present worldwide pandemic of corona-virus disease 2019 (COVID-19) has resulted in more than 20,439,814 cases and 744,385 deaths [13]. The causative agent of COVID-19 is a virus called SARS-CoV-2 since its single-stranded RNA genome is 82% identical to that of SARS-CoV [14,15,16].

SARS-CoV recognizes angiotensin-converting enzyme 2 (ACE2) [17] on the host cell membrane as a specific receptor using the spike (S) protein of the virus. The interaction of the viral S protein with the host cell receptor is followed by membrane fusion of the virus and host cell, which transports the virus RNA into the host cell. Thus, an agent such as a soluble ACE2, or an antibody to this protein, could be a possible inhibitor of the virus-cell interactions. The viral genome injected into the host cell is then translated and processed to virus-derived structural proteins, including spike (S), envelope (E), membrane (M), and nucleocapsid (N) proteins, as well as nonstructural proteins that are used for the construction of viral particles. Therefore, inhibition of the processing reaction, essential for the generation of viral proteins, is a promising approach for the suppression of viral proliferation. Papain-like protease (PL^pro^) and chymotrypsin-like protease (CL^pro^) are the essential proteases for the processing reaction; PL^pro^ cleaves the N-terminal region of the viral precursor protein at three sites whereas 3CL^pro^ cleaves the C-terminal region of the precursor protein at 11 sites. The features of the SARS-CoV 3CL^pro^, which cleaves the precursor protein at three times more sites than PL^pro^ and has no known homologs in the host cell [18,19], making it an ideal target for antiviral agents. In addition, the sequence of SARS-CoV-2 3CL^pro^ shares 96% homology with that of SARS-CoV 3CL^pro^, initially identified from the SARS causative coronavirus [20]. These findings indicate that the studies on the SARS-CoV 3CL^pro^ are robust bases for designing therapeutic agents for COVID-19.

In this short review article, the efforts to develop therapeutic agents for SARS focusing on the inhibitors of SARS-CoV 3CL^pro^ are described. Instead of an exhaustive survey of the inhibitors [21], we provide an overview of several typical inhibitors, and our recent efforts for the rational design of new scaffolds are discussed based on the inhibitory mechanism and structural interactions with SARS-CoV 3CL^pro^. First, the protein chemistry of the target enzyme, SARS-CoV 3CL^pro^, is described in brief, as a basis for the structural analyses of protease-inhibitor interactions.

## 2. SARS 3CL Protease

The 29.7 kb positive-strand RNA genome of SARS-CoV contains two open reading frames (ORFs 1a and 1b) encoding two large replicative polyproteins, pp1a (486 kDa) and pp1ab (790 kDa) [22,23]. The expression of the ORF1b-encoded region of pp1ab is involved in the ORF produced by ribosomal frameshifting into the –1 frame just upstream of the ORF1a translation termination codon. The pp1a and pp1ab polyproteins are processed by two cysteine proteases: papain like protease (PL^pro^) and 3C-like protease (3CL^pro^, also called main protease, M^pro^). The name 3C-like is derived from picornavirus 3C proteases having a similar substrate specificity, which is encoded on the noncapsid region 3C [24]. The SARS-CoV 3CL^pro^ cleaves 11 sites of the pp1ab [25] and is indispensable for viral replication, but is not found in the host cells, which makes the SARS-CoV 3CL^pro^ an ideal target for antiviral agents [18,19].

**Figure 1:**
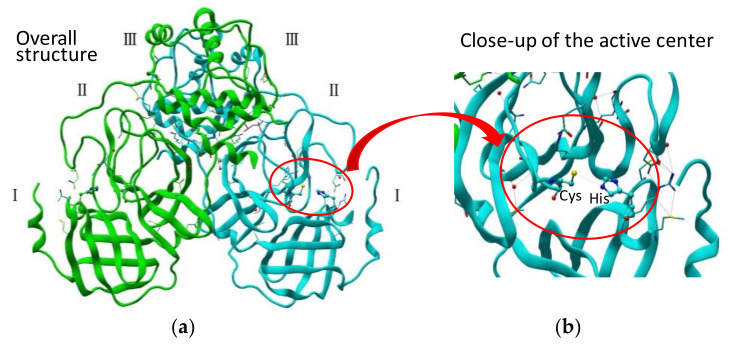
(**a**) Crystal structure of SARS-CoV 3CL^pro^ active dimer (PDB code 1Q2W). (**b**) Structure of the active center.

SARS-CoV 3CL^pro^ consists of 306 amino acid residues and contains the catalytic dyad defined by His41 and Cys145. The N-terminal part (1–184, domains I and II) is composed of a two-β-barrel fold forming the chymotrypsin-like architecture as in picornavirus 3C^pro^. The substrate-binding site is located in a cleft between these two domains. The C-terminal part (201–303, domain III), containing five α-helices, adopts a globular fold. Domain III, connected to domain II via a long loop, is a globular cluster of five helices, which is an essential architecture to hold the active dimer-structure of the protease (Figure 1a) [26]. Most CoV-derived 3CL proteases recognize a conserved (Leu/Ile)-Gln ↓ (Ser, Ala, or Gly) core sequence as a canonical sequence (the cleavage site is indicated by ↓) at the active center (Figure 1b) [27]. A comparison of the cleavage efficiencies of synthetic substrates containing the 11 cleavage sites of SARS 3CL^pro^ confirmed that the most suitable substrate was the N-terminal site of SARS 3CL^pro^ itself, suggesting that SARS 3CL^pro^ cleaves itself most efficiently. A study using a fluorescent-dodecapeptide as an experimental substrate also revealed a similar tendency of SARS-CoV 3CL^pro^ substrate recognition, as well as differences between SARS-CoV 3CL^pro^ and MERS-CoV 3CL^pro^ [28]. Moreover, recent studies on the SARS-CoV polyprotein processing using native mass spectrometry (MS) combined with collision-induced dissociation (CID) revealed a dynamic reaction, including substrate consumption, the rise and fall of intermediate products and complexation [29].

**Figure 2:**
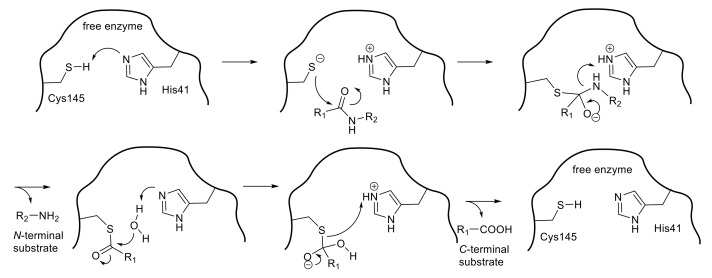
Hydrolysis of the substrate by thiol protease.

SARS 3CL^pro^ is a cysteine protease in which Cys145 and His41 form a catalytic dyad (Figure 2). The initial step of the hydrolysis is the deprotonation of Cys145-thiol by the imidazole group of His41 to increase the nucleophilicity of the thiol group, which then attacks the substrate carbonyl carbon. Therefore, SARS 3CL^pro^ shows the highest enzymatic activity at approximately pH 7, retaining the un-protonated imidazole form of His41. After the nucleophilic attack, the C-terminal substrate fragment is released from the enzyme, leaving a covalently modified enzyme by thioester formation between the enzyme thiol group and the carbonyl group at the substrate scissile site. The thioester is then hydrolyzed by the nucleophilic attack of a deprotonated water molecule, and the corresponding C-terminal fragment of the substrate is released to generate the free enzyme. Thus, compounds containing a functional group, a so-called “warhead” which interacts with the thiol group of Cys145, may be promising agents for inhibiting the catalytic potency of the cysteine protease.

The first step of our inhibitor studies on SARS-CoV 3CL^pro^ was the production of a mature protease on a scale of several milligrams per batch by a conventional expression procedure using *E. coli* to establish the assay protocol and crystallization procedure; however, the amount of the protease obtained from the initial trial was insufficient compared with the expected amount. Careful examination of our expression procedure to yield mature SARS-CoV 3CL^pro^ suggested that the mature 3CL^pro^ was somehow susceptible to degradation, particularly considering Arg188 located at the connecting loop between domains II and III. Therefore, a mutated protease R188I SARS-CoV 3CL^pro^, with an Ile instead of an Arg at position 188, was produced using *E. coli* to yield the expected amount with high homogeneity [30]. Of note, the mutation increased the stability and maintained almost the same three-dimensional structure (PDB code 3AW1) as that of native SARS-CoV 3CL^pro^. The use of the mutant R188I SARS-CoV 3CL^pro^ allowed the evaluation of enzymatic activity via conventional high-performance liquid chromatography (HPLC) without the need to use any specific substrate with fluorescent substituents.

## 3. Inhibitors of SARS-CoV 3CL^pro^

After the SARS-CoV pandemic in 2003, numerous studies have been conducted to identify inhibitors of SARS-CoV 3CL^pro^ [31,32,33,34]. These inhibitors are structurally classified into two types: peptide-mimetic inhibitors and nonpeptide small-molecule inhibitors. Based on the inhibitory mechanism, these inhibitors can also be classified into irreversible (those that form a covalent bond with 3CL^pro^), and reversible (noncovalent) inhibitors (those that compete with the substrate). A variety of first-generation inhibitors reported after the first outbreak of SARS-CoV provided valuable insights into further modifications through structure-based design. In the following sections, several typical inhibitory mechanisms, as well as our structural modification studies of peptide-mimetic to nonpeptide inhibitors, are described.

### 3.1. Peptide-Mimetic Inhibitors Containing a Michael Acceptor

A peptide-mimetic protease inhibitor generally contains a substrate-like sequence and a “warhead” interacting with the catalytic center of the target enzyme. In the inhibitors for SARS-CoV 3CL^pro^, the substrate-like sequence is designed by optimizing the specific interactions at the S1’ to S4 sites of the substrate. The warhead interacting with the active center thiol of SARS-CoV 3CL^pro^ should be an electrophilic functional group, considering the reaction mechanism of thiol protease described above. Among the various electrophilic functional groups, a Michael acceptor is one of the most commonly used warheads to effectively form a covalent bond by a nucleophilic attack with a thiol.

**Figure 3:**
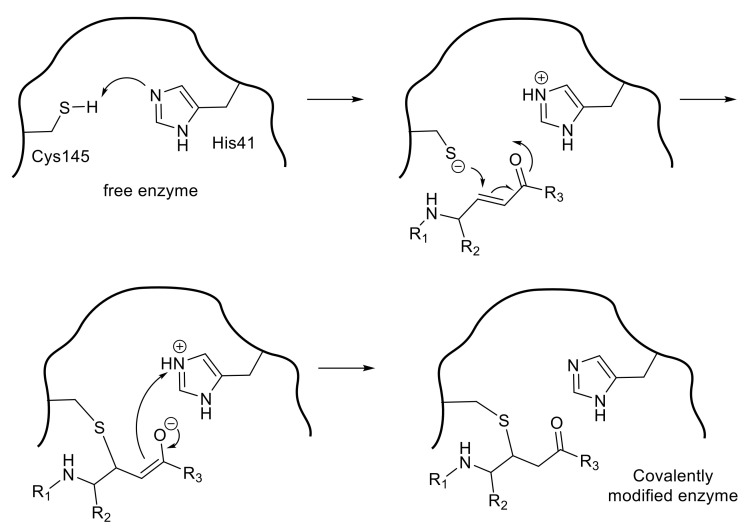
Inhibition of cysteine proteases by a Michael acceptor type compound.

A peptide-mimetic inhibitor containing a Michael acceptor involves the replacement of a substrate’s scissile amide bond with an appropriate Michael acceptor. Following the interaction with the active center of the SARS-CoV 3CL^pro^, the nucleophilic Cys145 thiolate generated by a proton-withdrawing effect caused by His41 at the catalytic dyad promotes a typical 1,4-addition to the α,β-unsaturated structure of the Michael acceptor (Figure 3). The resulting protonated His41 donates the proton to an unstable intermediate anion to form 3CL^pro^ covalently bound to the inhibitor. Thus, the Michael acceptor type compound acts as a type of suicide substrate to abolish the catalytic activity of the enzyme by covalent modification.

In a series of evaluations, using compound **1** targeting rhinovirus 3CL^pro^ as a starting compound, optimizations of the side-chain structures at P1’ to P4 sites of the SARS-CoV substrate were conducted to develop SARS-CoV 3CL^pro^ specific inhibitors [35,36,37] (Table 1). The interactions of the SARS-CoV 3CL^pro^ with some of these inhibitors were analyzed by X-ray crystallography (PDB codes 2ZU4 and 2ZU5), which confirmed that Cys145 sulfur and the α-carbon of the Michael acceptor at the P1’ site formed a covalent bond of 1.99 Å. Inhibitors with a five-membered lactam ring at the P1 site showed much stronger inhibitory activities than those with glutamine at the P1 site. For the P2 site substituent, a hydrophobic isopropyl substitute was preferred over a rigid and planar phenyl substitute (compounds **3** vs **2**), indicating that the S2 pocket of SARS-CoV 3CL^pro^ will accept rather large hydrophobic substituents. The P3 substituent is expected to be directed towards the bulk solvent and would have no interactions with the protease. Unexpectedly strong inhibitory activities of compounds **4** to **3** were probably due to shifting of the *N*-terminal substituent toward the P4 site by a neighboring bulky *tert*-butyl group inducing hydrophobic interactions with 3CL^pro^. In another series of studies [38], the effect of methylene insertion between the reactive Michael acceptor and the sessile site was investigated. The results clearly showed that no elongation of the Michael acceptor structure toward the prime site was tolerated (Table 2), which strongly suggests strict recognition of the prime-site structure.

### 3.2. Peptides with Halomethyl Ketone

**Figure 4:**
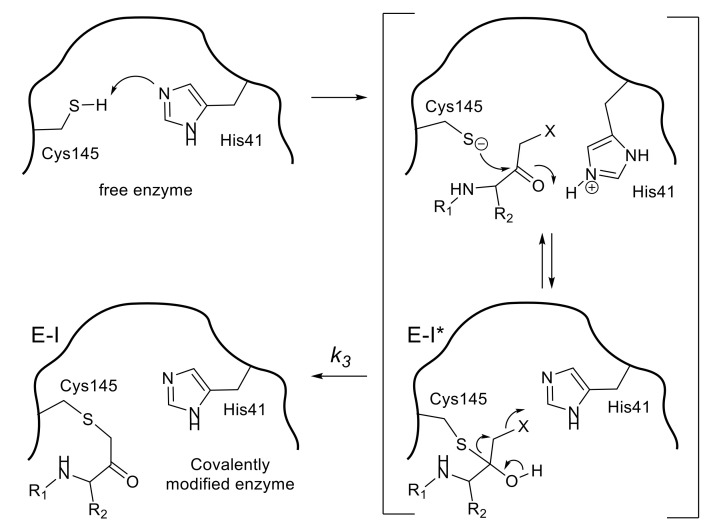
A possible mechanism for the inactivation by a halomethyl ketone inhibitor.

Halomethyl ketone groups can form a covalent bond by an apparent alkylation caused by a thiolate anion, because the halomethyl group makes the adjacent ketone group more susceptible to a nucleophilic attack. The initial nucleophilic attack of a thiolate of Cys145 of 3CL^pro^ toward the carbonyl group of the halomethyl ketone leads to the reversible formation of a tetrahedral thiohemiketal that resembles the intermediate configuration in substrate cleavage (Figure 4). The subsequent intramolecular rearrangement leads to the final product, a covalently modified inactive enzyme. A direct mechanism, in which the thiolate ion directly attacks the halomethyl carbonyl group to yield the alkylated enzyme, is less conceivable than the above intramolecular rearrangement pathway based on the experimental kinetics data.

An initial study of a series of inhibitors containing P1 site *N,N*-dimethyl glutaminyl fluoromethyl ketone combined with different P2 site substituents revealed two possibilities. One was the effectiveness of a halogenated methyl group as a warhead, and another was the remarkable contribution of the P2 site substituent on the inhibitory activity, probably due to the specific interactions with 3CL^pro^ (Table 3a) [39]. The antiviral activity assessed by cytopathic effect (CPE) inhibition in SARS-CoV infected Vero cell cultures, revealed that compound **10**, with P2-Leu, can protect the cells against SARS-CoV infection with an EC_50_ of 2.5 μM, as well as the low toxicity in mice. The P2 Leu of **10** can be replaced by Ile or Val, resulting in slightly lower EC_50_ values (compounds **11** and **12**). In addition, these active compounds were inactive against rhinovirus type-2 in a cell-based assay, suggesting that they are specific for SARS-CoV. In contrast, removal of these P2-site substituents abolished the inhibitory activity (compound **13**), indicating that the hydrophobic interactions at the P2 site were essential for potent inhibition.

Studies on another series of halomethyl ketone type inhibitors revealed additional possibilities regarding P1 site substituent and the inhibitory reaction mechanism (Table 3b) [40]. The results indicated that hydrophobic P1 substituents, such as simple aromatic groups (phenyl and naphthyl) or an aliphatic bulky group, were tolerated as those of a rather complexed lactam ring, keto-glutamine analogs, or an α,β-unsaturated ester structure. These data also indicate that the corresponding S1 pocket of the SARS-CoV 3CL^pro^ might accept a simple ring structure containing heteroatoms at this specific interaction site, which provides a clue to our design of a potent substrate-based inhibitor described later in this review.

Additional information obtained from these series of inhibitors is the effect of the halogen atom in the halomethyl ketone group. The rate of the final irreversible step of the inhibition pathway (*k_3_* in Figure 4) is directly related to the acceptability of a nucleophilic attack for the eventual alkylation. The values of *k_3_* of compound **14**–**16** (2.8 × 10^−2^/s–1.5 × 10^−2^/s) indicated that the chloromethyl ketone was effective as a warhead, although the *k_3_* value varied by two-fold depending on the bulkiness of the P1 site substituent. In contrast, the *k_3_* value of bromomethyl ketone inhibitor **17** was too small to be measured, indicating that the irreversible step was very slow. Indeed, inhibitor **17** behaved as a reversible inhibitor for several hours and irreversible inhibition of the enzyme activity was only noticed after a 12 h incubation with inhibitor **17**. Thus, a time-dependent bimodal mode of inhibition for this inhibitor **17** was suggested, in which the initial formation of a reversible complex (E-I*) occurred followed by rearrangement to an irreversible complex (E-I) after at least 6 h incubation. In the crystal structure of SARS-CoV 3CL^pro^ complexed with inhibitor **17** (PDB code 3D62), a thioether bond (1.7 Å) between the carbon originally bound to bromine and the sulfur atom of Cys145 was clearly detected supporting the above bimodal pathway.

### 3.3. Peptides with Trifluoromethyl Ketone and Related Electrophilic Substituent

**Figure 5:**
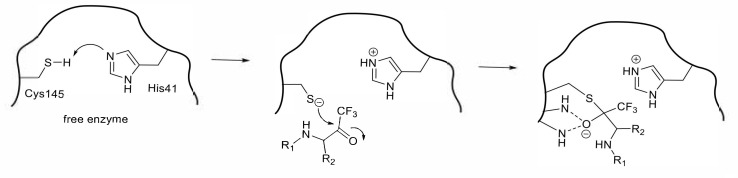
Proposed mechanism of inhibition by a trifluoromethyl ketone compound.

A trifluoromethyl group is another electron-withdrawing group that makes a neighboring carbonyl group susceptible to nucleophilic attack. Initial studies of trifluoromethyl ketone type inhibitors (Table 4) yielded in an *N*-protected tetrapeptide compound **22** (IC_50_ = 10 μM) [41]. A Lineweaver–Burk plot obtained from the inhibition reaction by **22** confirmed a competitive inhibition mode in the initial 4 h reaction, whereas a time-dependent decrease in the enzymatic activity as a function of the inhibitor concentration was observed in the prolonged reaction. The slow formation of a covalent adduct caused by the nucleophilic attack of the thiol on the carbonyl carbon was assumed to provide a rational explanation of the kinetic results (Figure 5). Computational molecular modeling also rationalized the covalent bond formation, illustrating a transition state mimic in the substrate cleavage by SARS-CoV 3CL^pro^.

**Figure 6:**
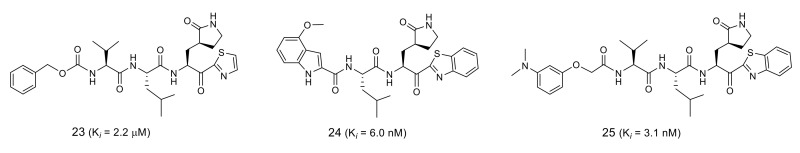
Inhibition with peptides having thiazolyl ketone warhead.

Further studies on the related groups using the trifluoromethyl equivalent revealed that a thiazolyl ketone group could increase the inhibitory activity ten-fold due to its high electrophilicity (Figure 6). Structural optimization at the P4 site combined with a benzothiazole warhead yielded inhibitors **24** and **25**, which both had IC_50_ values in the low nanomolar range [42,43].

### 3.4. Nitrile-Based Peptide-Mimetic Inhibitors

**Figure 7:**
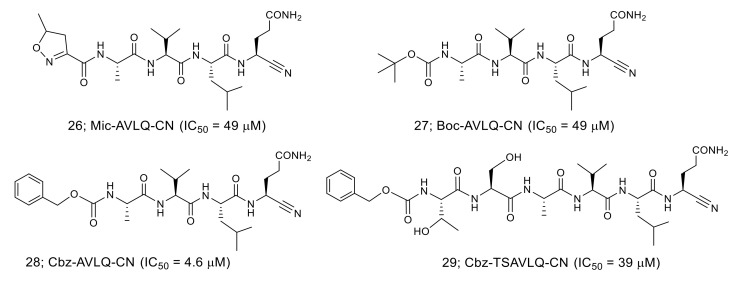
Structures and IC_50_ values of the nitrile-based inhibitors against SARS-CoV 3CL^pro^.

A nitrile group used as a warhead in an anti-diabetes DPP4 inhibitor is another functional group that has been incorporated in the inhibitor for SARS-CoV 3CL^pro^ (Figure 7). Among the nitrile-based tetrapeptide inhibitors with different *N*-protecting groups (Mic:5-methylisoxazole-3-carboxyl, Boc: *tert*-butyloxycarbonyl, and Cbz: carboxybenzyl), Cbz-AVLQ-CN **28** was ten-times more potent (IC_50_ = 4.6 μM) than the other inhibitors [44]. Interestingly, the longer Cbz-hexapeptide inhibitor, Cbz-TSAVLQ-CN, was less active than the tetrapeptide inhibitor **28**. The results suggest that the Cbz group of **28** might function as the P4 substituent interacting at the S4 pocket of SARS-CoV 3CL^pro^.

**Figure 8:**
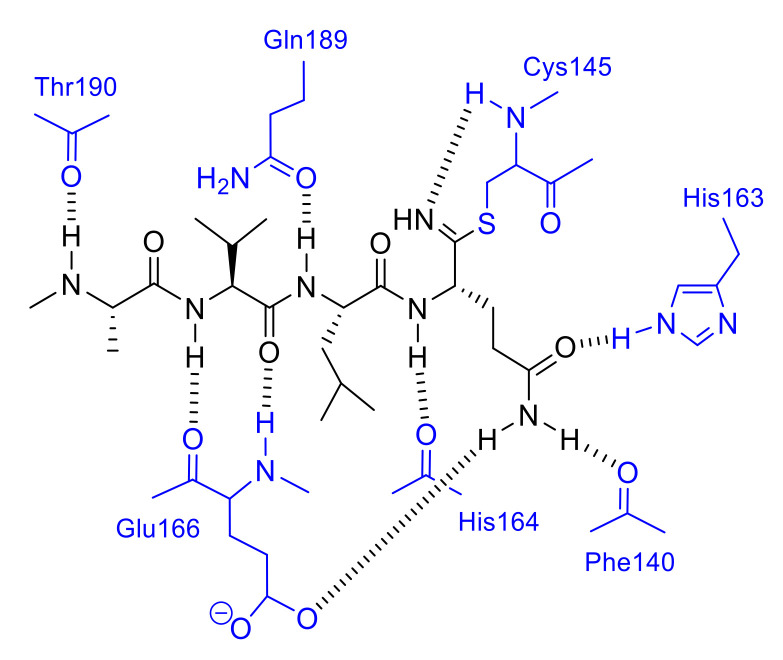
Interactions of the nitrile-based inhibitor with SARS-CoV 3CL^pro^.

The crystal structures of the SARS-CoV 3CL^pro^ in complex with the nitrile-based inhibitor (PDB codes 3VB7, 3VB4, 3VB5, and 3VB6) demonstrated that the inhibitor was covalently bonded to the thiol group of Cys145 via the nitrile warhead (Figure 8). In addition, the tetrapeptide inhibitor Cbz-AVLQ-CN inhibited 3CL^pro^ from human coronavirus strains such as 229E (IC_50_ = 2.3 μM), NL63 (IC_50_ = 2.8 μM), OC43 (IC_50_ = 1.6 μM), and HKU1 (IC_50_ = 1.3 μM). In contrast, the same inhibitor had no observable inhibitory effect on caspases, which are common host cells proteins and effectors of apoptosis. These results suggest that the nitrile-based inhibitor is a specific and broad-spectrum inhibitor for coronavirus 3CL^pro^.

### 3.5. Peptide Aldehydes

**Figure 9:**
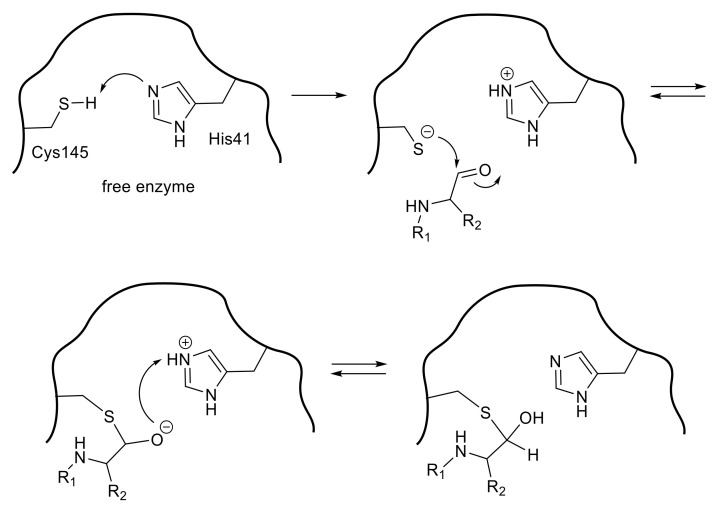
Nucleophilic addition reaction to peptide aldehydes.

Peptide aldehyde with a substrate-like sequence has the potency to be an effective inhibitor for thiol proteases as an aldehyde group is another reactive electrophilic group involved in the nucleophilic addition of a thiol to yield hemithioacetal (Figure 9).

**Figure 10:**
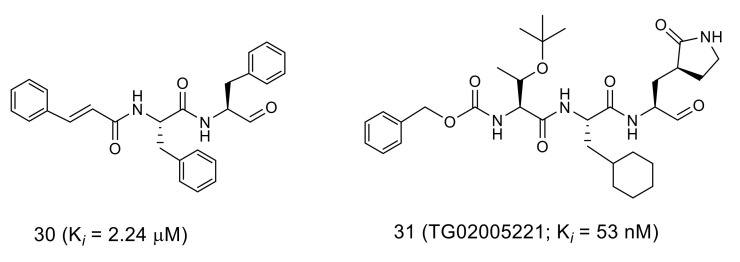
Structures of peptide aldehydes **30** and **31**.

Initial studies on aldehyde-type inhibitors led to a potent inhibitor **31** (Figure 10; *K_i_* = 53 nM) through the extensive structural optimization of a prototype inhibitor **30** based on the structural evaluation of the SARS-CoV 3CL^pro^ complexed with inhibitor **30** (PDB code 3SN8) [38,45]. The analyses of the crystal structure of SARS-CoV 3CL^pro^ complexed with the resulting inhibitor **31** (PDB code 2GX4) revealed that the distance of the thiol sulfur atom of Cys145 and the carbonyl carbon of the aldehyde was 1.24 Å, an equivalent distance to a covalent C-S bond. In addition, an oxyanion hole is expected to be formed by the coordination of the N-H of Cys145 and Gly143 at the S1 pocket, which would stabilize a tetrahedral intermediate of the nucleophilic addition reaction. The P1 and P4 substituents of inhibitor **31** are held by hydrogen bonds at the corresponding S1 and S4 pockets of SARS-CoV 3CL^pro^, and the P2 site cyclohexyl group formed hydrophobic interactions at the S2 pocket of the protease.

**Figure 11:**
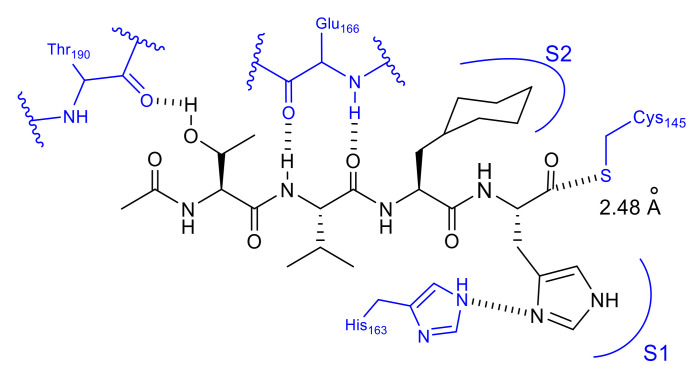
Interactions of inhibitor 37 with R188I SARS-CoV 3CL^pro^.

In our own studies on a series of substrate-based peptide aldehyde inhibitors, optimization of the P1 substituent was first conducted as the scissile site substituent generally shows the main influence on the enzyme specificity. As summarized in Table 5, a series of replacement functional groups at the P1 site of the substrate-based inhibitor **32** revealed imidazole was the most effective substituent (inhibitor **35**, IC_50_ = 5.7 μM), which showed more than six-fold stronger inhibitory activity compared with **32** (IC_50_ = 37 μM). Further structural analyses of the R188I SARS-CoV 3CL^pro^ complexed with inhibitor **35** (PDB code 3AW0) revealed several noteworthy information regarding the interactions with 3CL^pro^: (i) the large hydrophobic S2 pocket was not fully occupied; (ii) the P3 substituent was directed outward of 3CL^pro^, resulting in no interactions with the protease; (iii) the S4 pocket was not fully occupied, and additional interactions via hydrogen bonds appeared feasible, and iv) the P5 substituent extended outside of 3CL^pro^ and is not involved in the interactions with the 3CL^pro^. Further structure optimization based on these inspections provided a potent tetra-peptide aldehyde inhibitor **37** (IC_50_ = 98 nM) [40]. The X-ray crystal structure analysis of R188I SARS-CoV 3CL^pro^ complexed with **37** (PDB code 3ATW) confirmed the expected tight hydrophobic interactions formed by the P2 cyclohexyl group and hydrogen-bond interaction at the P4 site Thr (Figure 11).

In these X-ray structural analyses, the distance between the carbonyl carbon of the aldehyde and the thiol sulfur of the Cys145 was 2.30 Å (Figure 11), and the electron density of the aldehyde group could be fitted to a carbonyl sp^2^ carbon. These findings suggest that the aldehyde of inhibitor **37** interacts with the thiol of 3CL^pro^ noncovalently. In addition, pre-incubation of inhibitor **37** with 3CL^pro^ prior to the addition of the substrate caused no change in the IC_50_ value compared with that obtained by simultaneous mixing of the inhibitor **37**, 3CL^pro^, and the substrate, which suggests that no stable covalent bonds were formed between the inhibitor **37** and 3CL^pro^. Kinetic data obtained from Lineweaver-Burk plots confirmed that aldehyde type inhibitor **37** functions as a competitive inhibitor without forming an irreversible covalent bond.

### 3.6. Rational Design of SARS-CoV 3CL^pro^ Inhibitors Based on Structural Analyses

Although the substrate-based inhibitors described above showed strong inhibitory potency, the in vivo instability of these peptide-based compound was expected to be a major drawback preventing use as an oral therapeutic agent. The development of nonpeptide small-molecular SARS-CoV 3CL^pro^ inhibitors would be an approach to overcome these drawbacks. To this end, three different procedures have been generally employed: evaluation of natural products, high-throughput screening of synthetic compound libraries, and the rational design of a new scaffold of inhibitor. Although a few examples of the inhibitors derived from natural products [46,47,48] or virtual or high-throughput screening [49,50,51,52,53] have been reported, examples of the structure-based rational design of a new scaffold are very limited, and the inhibitory activities are still moderate. Nevertheless, the rational approach contributes largely to the design of a variety of novel scaffolds with high potential as a therapeutic agent for SARS-CoV -related diseases. In the following section, two rational approaches for the design of nonpeptide inhibitors derived from a peptide aldehyde are described.

**Figure 12:**
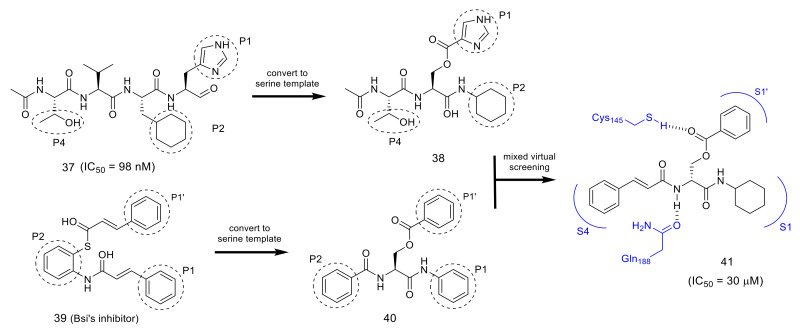
Design of the serine derivative as a nonpeptide inhibitor for SARS-CoV 3CL^pro.^

As an approach for nonpeptide inhibitor, serine was selected as an attractive scaffold as this commercially available proteinogenic amino acid has three functional groups available for modification: an alcohol, an amino, and a carboxylic acid group [54]. Each group of the serine can be orthogonally modified, which makes it feasible to introduce respective substituents corresponding to the P1 to P4 sites into the scaffold independently. As the parent inhibitor to be modified, the highly potent peptide aldehyde **37** was used and substituents at the P1, P2, and P4 sites, as those are the sites that interact closely with the respective mode of SARS-CoV 3CL^pro^, were selected as the substituents to be introduced on the serine scaffold (Figure 12, compound **38**). An energetically favorable conformation mimicking the parent inhibitor **37** was then sought by molecular mechanic’s calculation performed with SPARTAN combined with docking simulations by GOLD. 

The results of the initial trial suggested an unexpected positioning of the substituent, in which the cyclohexyl group at the serine amid carbonyl occupied the S1 pocket instead of the expected S2 pocket of 3CL^pro^. Similarly, it was reported by Bai et al. that a cinnamoyl derivative was expected to interact with 3CL^pro^ at the S1′, S1, and S2 pockets (Figure 12, compound **39**) based on simulations using Autodock 3.0. Considering the contrasting results obtained from both simulations, a hybrid scaffold was designed combining Bai’s derivative and the serine derivative (Figure 12). Extensive SAR studies of the hybrid scaffold focusing on the P1’ and P4 substituents resulted in an optimized compound **41** (IC_50_ = 30 μM) as a novel small molecule inhibitor derived from serine. Docking simulations of compound **41** with SARS-CoV 3CL^pro^ confirmed the expected interactions at the S1’, S1, and S4 pockets (Figure 12).

**Figure 13:**
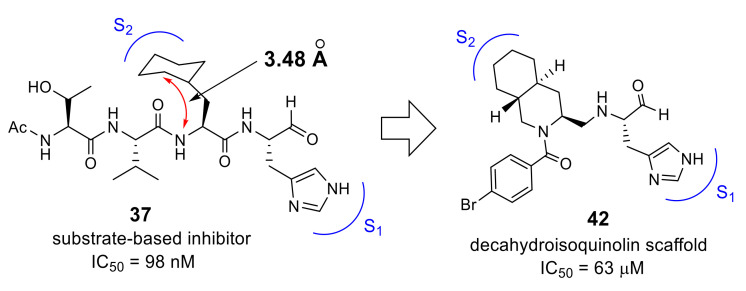
Design of a decahydroisoquinoline scaffold.

Another approach starting from the same peptide inhibitor **37** was based on closer inspection of the interactions with the SARS 3CL^pro^ [55]. Previous analyses of the crystal structure of the SARS-CoV 3CL^pro^ complexed with inhibitor **37** (PDB code 3ATW) revealed that a cyclohexyl substituent of cyclohexylalanine (Cha) at the P2 site was well packed in the hydrophobic S2 pocket and formed a critical interaction to make **37** a highly potent inhibitor. Detailed structural evaluation of this hydrophobic pocket revealed that the P2 site cyclohexyl ring was rather close to the peptide backbone. In the crystal structure, the distance of the position 2 carbon (C2) of the cyclohexyl ring to the α-amide nitrogen of Cha at the P2 site was estimated to be 3.48 Å. The distance is approximately equal to the sum of two covalent bonds, and the connection of the two atoms by a methylene linker appears to be feasible, yielding a novel fused ring structure, a decahydroisoquinoline scaffold, that acts as a hydrophobic substituent at the P2 position (Figure 13). In addition, this fused ring scaffold can be a core scaffold to arrange the P1 site imidazole and active site functional aldehyde at the required positions. The acyl substituent on the nitrogen atom in the decahydroisoquinoline scaffold is expected to be a new substituent providing additional interactions with 3CL^pro^.

**Figure 14:**
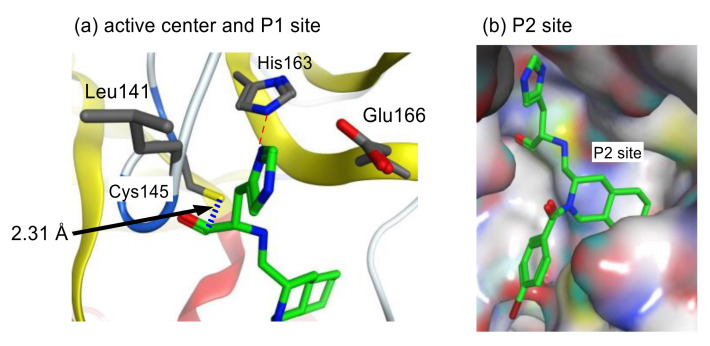
Interactions of inhibitor **42** at the active center (**a**) and P2 site (**b**) of SARS-CoV 3CL^pro^.

To assess the validity of the above design, all possible configurations at the fused ring of the decahydroisoqunolin scaffold were separately synthesized by a combination of enantiomer resolution and Pd-catalyzed stereoselective cyclization reaction. Each synthesized derivative showed moderate but clear inhibitory activity, although the potency was different depending on the configuration. A specific configuration of inhibitor **42** (Figure 13) was the most effective configuration, confirming the utility of the decahydroisoquinoline scaffold as a hydrophobic core. In addition, the acyl group on the nitrogen atom of the scaffold showed a limited effect on the inhibitory activity. X-ray crystal analyses of SARS-CoV 3CL^pro^ complexed with inhibitor **42** and related compounds (PDB codes 4TWW, 4TWY, and 4WY3) rationalized the effective interactions causing these differences. The distance between the carbonyl carbon of the aldehyde of **42** and thiol sulfur of Cys145 was 2.43 Å, suggesting that the decahydroisoquinoline inhibitor was a competitive inhibitor like the parent peptide inhibitor **37** (Figure 14). The P1 site imidazole of **42** located at the S1 pocket and the nitrogen atom of the imidazole formed hydrogen bonds, similar to the parent inhibitor **37**. The decahydroisoquinoline scaffold of **42** adopted a *trans*-fused configuration and occupied most of the S2 pocket of 3CL^pro^ directing the P1 site imidazole and warhead aldehyde into the active center. In contrast, the acyl substituent bound to the nitrogen of the fused ring was located on the surface of 3CL^pro^, where an additional interaction with the protease might be possible.

**Figure 15:**
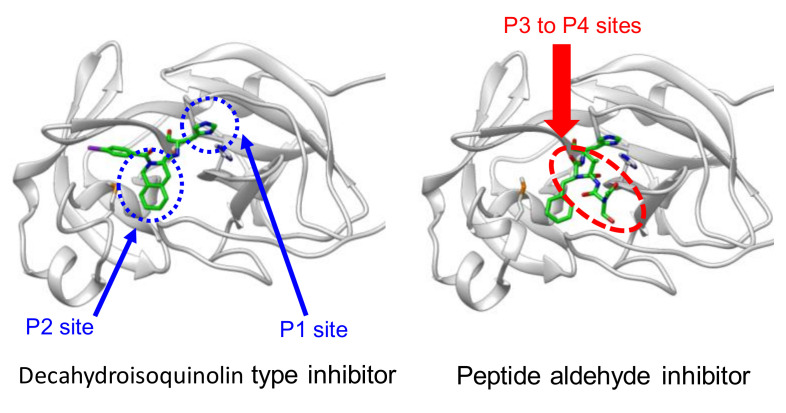
Comparison of the X-ray crystal structure of SARS-CoV 3CL^pro^ complexed with decahydroisoquinoline-type inhibitor **42** (PDB code 4TWW) and the peptide aldehyde inhibitor **37** (PDB code 3ATW).

To improve the moderate inhibitory activity of above decahydroisoquinoline-type inhibitor **42**, the overall interaction mode of **42** was compared with that of the parent peptide inhibitor **37**. An overlay of both interaction modes suggested that the nonprime site interactions of the parent inhibitor **37** were missing in the interaction mode of the decahydroisoquinoline-type inhibitor **42** (Figure 15).

**Figure 16:**
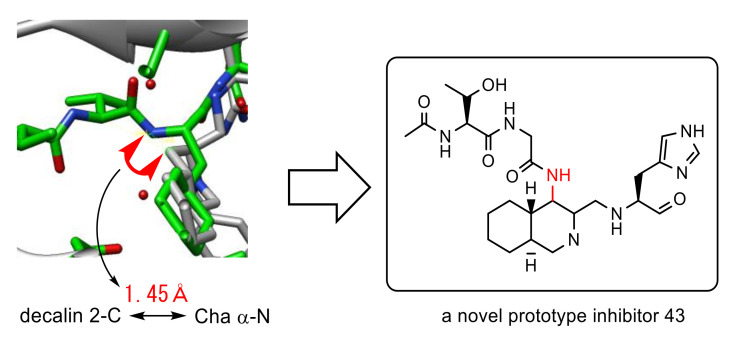
Design of a novel prototype nonpeptide inhibitor.

A detailed evaluation of the overlay of above both inhibitors complexed with 3CL^pro^ revealed that the distance between the α-nitrogen of Cha in the peptide aldehyde and 4-position of the decahydroisoquinoline scaffold was estimated to be 1.45 Å, a distance equivalent to a covalent bond. Thus, a dipeptide unit as a nonprime site substitute was introduced at the 4-position of the decahydroisoquinoline scaffold to yield a novel prototype inhibitor **43** (Figure 16) [56]. The synthesized inhibitor **43** showed 2.4 times greater inhibitory activity (IC_50_ = 26 μM) than the initial decahydroisoquinoline-type inhibitor **42**, which strongly suggested the positive effect of the 4-position substituent, probably through interaction with the nonprime site pocket of 3CL^pro^. Structural optimization of this nonprime site substituent should be the next step to create a novel lead structure of nonpeptide small-molecule inhibitors based on a rational design.

## 4. Conclusions

The inhibitor design briefly surveyed in this short review is a potential starting point for the development of anti-SARS-CoV agents; of note, most inhibitors described in this review have inhibitory potency against CoV as well as good physicochemical and pharmacodynamics properties necessary for in vivo use. Indeed, the inhibitory potencies against CoV in cells were confirmed for several compounds, including peptide-based inhibitors and small-molecule inhibitors. The low toxicity for cells was also examined for a few compounds, although further in vivo studies are required. Reevaluation of natural products such as flavonoids or Indian medicinal plants is an alternative approach to design clinically useful inhibitors for SARS-CoV 3CL^pro^ [57,58]. Enzymatic evaluations of the 3CL protease of SARS and MERS is another base for the development of therapeutic agents for SARS related respiratory diseases such as COVID-19. Recent studies on the catalytic mechanism of the SARS-CoV 3CL^pro^ and MERS-CoV 3CL^pro^ revealed detailed insights regarding the difference in catalytic efficiencies between 3CL^pro^ from SARS-CoV and MERS-CoV, and identified a potential allosteric site for inhibitor design.^30^ These findings should contribute to the development of therapeutic agents based on SARS-CoV-2 3CL^pro^. In addition, the crystal structure of SARS-CoV-2 3CL^pro^ revealed the potential effectiveness of an inhibitor designed on the basis of SARS-CoV 3CL^pro^; of notes, this may be another basis for the development of therapeutic agents for COVID-19 [59]. The rational design of a novel scaffold starting from a peptide-based inhibitor discussed in this review would be an alternative way to design novel cysteine protease inhibitors. Although compounds showing antiviral activity can be discovered by screening a library composed of approved drugs or therapeutics in clinical development, as used in the present measures devised to combat COVID 19, the development of novel and specific anti-SARS CoV inhibitors based on the achievements described in this review should be an alternative approach to consider, in the context of the treatment of SARS-related infectious diseases. Additionally, polymerase inhibitors and others should be considered, to target different vital routes, and effectively combat SARS-CoV-2 since multiple targets are useful to avoid resistance.

## Figures and Tables

**Table 1:**
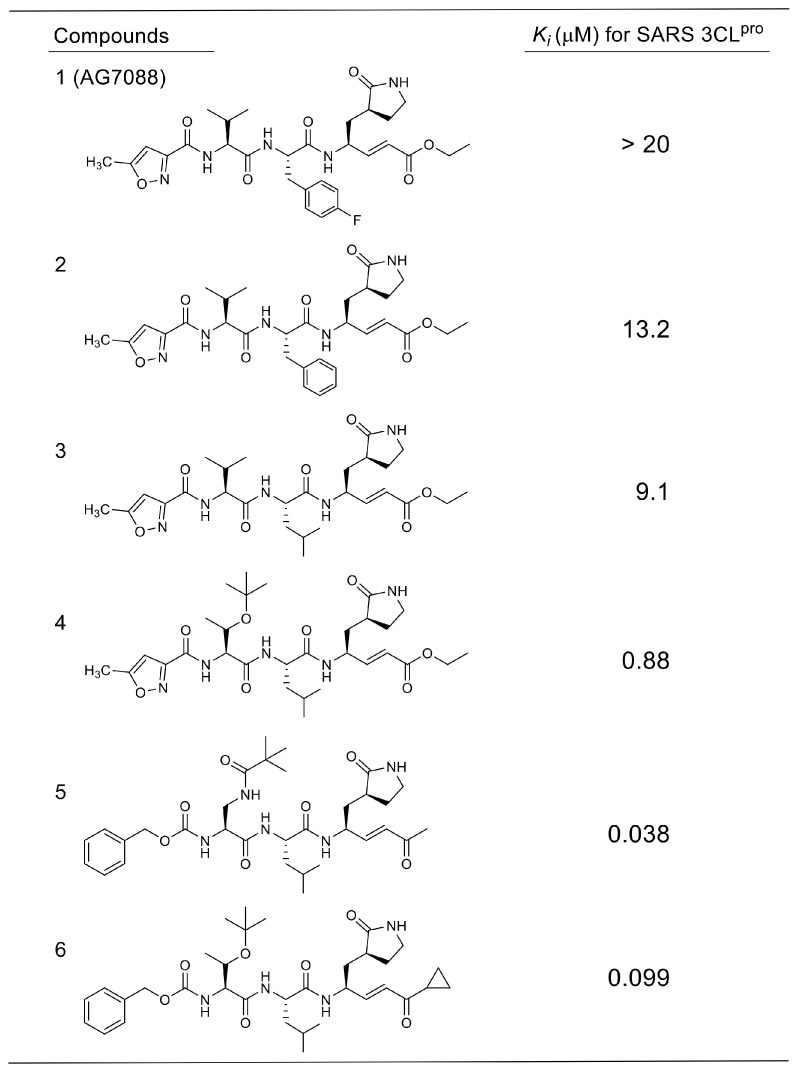
Inhibitory activities of Michael type inhibitors against 3CL^pro^.

**Table 2:**
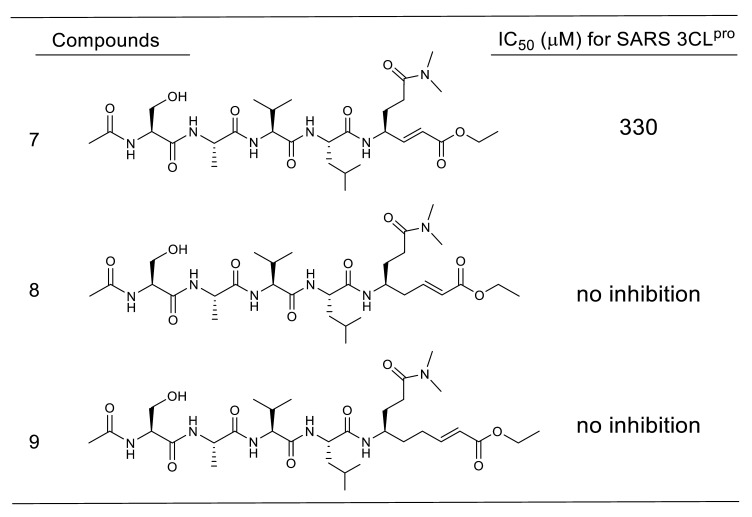
Effect of the methylene linker between the Michael acceptor and the sessile site.

**Table 3:**
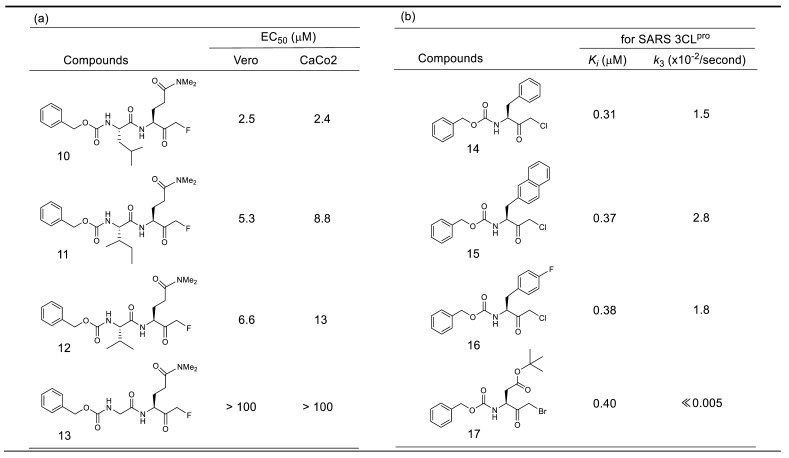
Inhibitory activities of halomethyl ketone type inhibitors against 3CL^pro^.

**Table 4:**
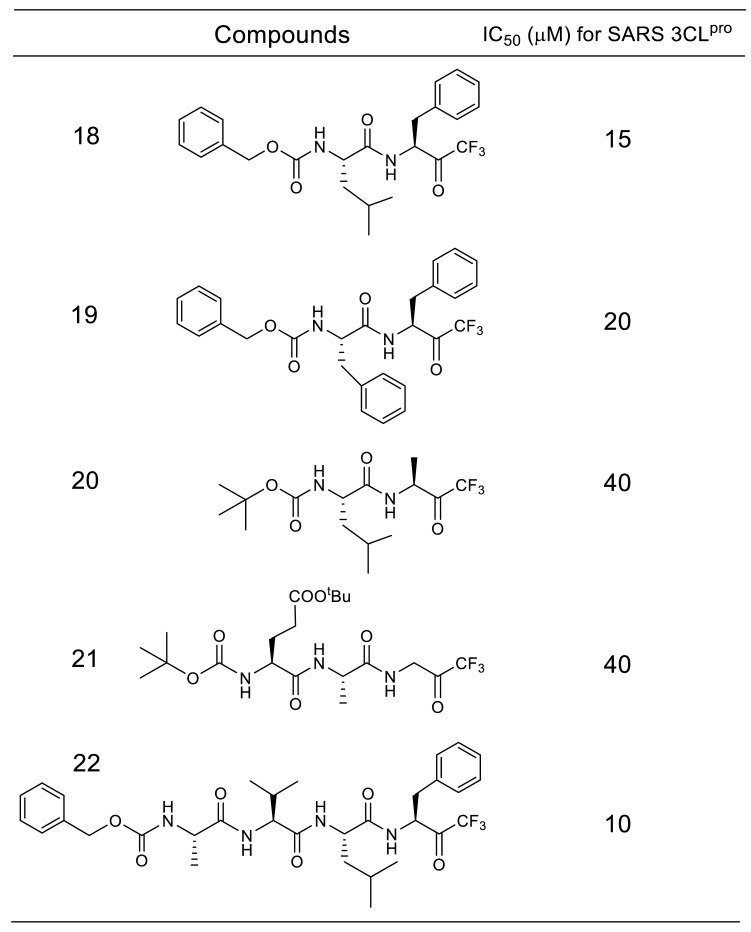
Inhibitory activity of substrate-based trifluoromethyl ketone compounds against SARS-CoV 3CL^pro^.

**Table 5:**
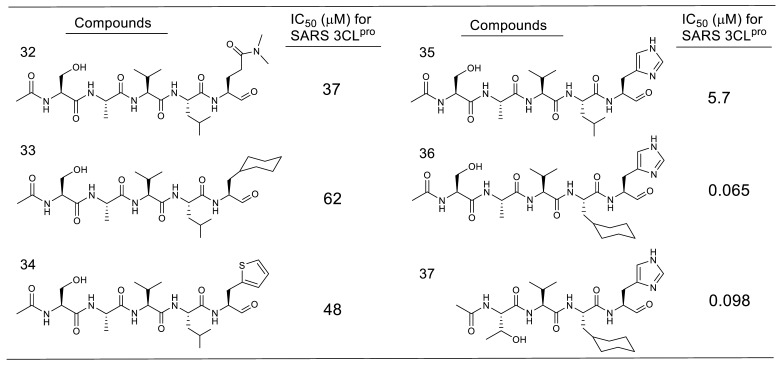
Optimization of a series of substrate-based peptide aldehyde inhibitors.
